# Metabolomic Characteristics of Cecum Contents in High-Fat-Diet-Induced Obese Mice Intervened with Different Fibers

**DOI:** 10.3390/foods12071403

**Published:** 2023-03-26

**Authors:** Qian Zhang, Jinhua Cheng, Xiaole Jiang, Junni Tang, Chenglin Zhu, Hong Chen, Luca Laghi

**Affiliations:** 1 College of Food Science and Technology, Southwest Minzu University, Chengdu 610041, China; 2 College of Food Science, Sichuan Agricultural University, Ya’an 625014, China; 3 College of Chemistry and Environment, Southwest Minzu University, Chengdu 610041, China; 4 Department of Agricultural and Food Sciences, University of Bologna, 47521 Cesena, Italy

**Keywords:** obesity, fibers, cecum contents, metabolome, ^1^ H-NMR

## Abstract

The aim of this study was to demonstrate the effect of single or mixed fibers (arabinoxylan, β-glucan, xyloglucan, and inulin) on the metabolome of cecum content in mice with obesity caused by a high-fat diet. Twenty-eight six-week-old male mice were divided randomly into seven groups (n = 4/group), including a normal-diet group (CON), a high-fat-diet group (HFD), and groups with the same high-fat diet but supplemented with arabinoxylan (HFAX), arabinoxylan + β-glucan (HFAβ), arabinoxylan + xyloglucan (HFAG), xyloglucan (HFXG), and xyloglucan + inulin (HFXI). A total of 66 molecules were identified and quantified in cecum content by proton nuclear magnetic resonance (^1^ H-NMR). The metabolomic profiles combined with statistical analysis revealed compounds distinguishing the control group from those supplemented with fibers. In detail, a high-fat diet could significantly elevate the concentrations of acetone and methionine (*p* < 0.05) while decreasing the levels of methanol, arabinose, acetate, and 3-hydroxyphenylacetate (*p* < 0.05) in the cecum contents of mice. Compared to HFD, the supplementation caused higher levels of fumarate and hypoxanthine (*p* < 0.05) and lower levels of phenylacetate, acetate, fucose, formate, proline, betaine, and trimethylamine N-oxide (TMAO) (*p* < 0.05). An enrichment analysis highlighted that the pathways mainly altered were amino sugar metabolism, aspartate metabolism, and arginine and proline metabolism. In conclusion, non-starch polysaccharide (NSP) supplementation could change the metabolomic profiles of cecum contents in obese mice as a result of a high-fat diet. Moreover, mixed NSPs exhibited more beneficial effects than singular form on gut metabolism.

## 1. Introduction

In recent years, obesity has become a major global public health issue, which has attracted widespread attention [1]. Obesity is an important risk factor for a series of metabolic diseases, such as diabetes, hypertension, hyperlipidemia, and cardiovascular and cerebrovascular diseases [2]. One of the main reasons causing obesity is the excessive consumption of high-fat foods. Therefore, proper dietary modification and effective control of energy intake and consumption are important for the prevention and treatment of obesity and its complications. In mice models, non-starch polysaccharides (NSPs) have been shown to exhibit various biological activities protecting mice from high-fat-diet-induced lipid derangements, inflammation, endotoxemia, and gut bacterial dysbiosis [3]. In detail, NSPs are broken down to short-chain fatty acids (SCFAs) by enzymes produced by gut microbiota, and then, SCFAs are able to facilitate the growth and reproduction of beneficial spices of gut microbiota [4,5]. Chen et al. found that NSPs administration could increase the abundance of *Lactobacillus* and *Bifidobacterium* and decrease the abundance of typical pathogenic microbiota (*Desulfovibrio* and *Proteobacteria*) [6].

Arabinoxylan (AX), which is one of the main NSPs in cereal, can help to prevent type 2 diabetes by regulating gut microbiota as well as metabolites. In detail, supplementation with AX has been shown to promote fiber-degrading bacteria, increase SCFAs, as well as to decrease opportunistic pathogens. Moreover, AX administration decreased the concentrations of 12α-hydroxylated bile acids and increased the levels of equol, indolepropionate, and eicosadienoic acid [7]. Glucan is a highly bioactive NSP, which includes xyloglucan (XG) and β-glucan (BG) [8]. XG is a major structural polysaccharide in the primary cell walls of higher plants, and it improves glycometabolism by interacting with gut microbiota [9]. BG is found in the cell walls of oat and barley endosperms. It can reduce weight and provide a feeling of satiety through the promotion of plasma glucagon-like peptide-1 (GLP-1) and peptide-YY (PYY) expression, which reduces appetite and counteracts high-fat-diet-induced fat production and weight gain [10]. Additionally, BG intake increases the abundance of *Bifidobacterium* and *Lactobacillus*, which are fermented by gut microorganisms into SCFAs, thus improving the metabolic situation [11,12]. Therefore, both XG and BG are now widely marketed as bioactive compounds with the potential to improve health [13]. Inulin is a prebiotic able to influence microbial composition and activity through tricarboxylic acid cycle (TCA cycle), with positive consequences mainly on SCFAs generation [14,15]. In addition, Li et al. found that inulin had beneficial effects on the abnormal liver lipid metabolism associated with diabetes mellitus type 2 (T2DM) [16]. Furthermore, our previous studies found that single or mixed fibers (AX, XG, BG, and inulin) could activate farnesoid X receptor (*Fxr*) and G-protein-coupled receptor 5 (*Tgr5*), decreasing bile acid levels from the liver of obese mice with high induced fat, in turn reducing liver damage. In line with the published studies, the concentration in the gut of beneficial microorganisms was found to be increased, mainly pertaining to the *Bacteroidetes* and *Akkermansia* species [6,17,18]. Although the beneficial effects of fibers are well documented in a series of publications, the detailed mechanisms of their regulation are still not fully understood, especially from a metabolomic point of view.

As the successor of genomics, transcriptomics, and proteomics, metabolomics shifts the focus from genes to small molecules, organically linking cells, tissues, and the whole process of an organism [19,20,21] Until now, metabolomics has been widely used to systematically analyze the dynamic metabolic changes in vivo induced by a high-fat diet through multiple biological substrates, such as serum [22,23], plasma [24,25], and urine [25,26]. In terms of the approaches for metabolomic analysis, ^1^ H-NMR spectroscopy has been widely applied, granted by its simple sample preparation procedures, its intrinsically non-invasive and reliable quantitative features, and its high reproducibility and superb instrument stability, which counterbalance its lower sensitivity than other platforms, such as GC-MS [14]. Up to now, metabolomics through ^1^ H-NMR platform has been adapted for in-depth obtaining the metabolic profiles of cecum contents in numerous remarkable publications, specifically powerful in identifying molecules linked with gut metabolism. For instance, Lanng et al. attempted to investigate the changes in gut metabolome related to the partial substitution of meat with insect (*Alphitobius diaperinus*) in a carnivore diet. The result showed that insect diet was pertaining to a higher amount of choline and acetate as well as the amino acids (for instance, aspartate, methionine, and glutamate) and the aromatic amino acids (such as tyrosine and phenylalanine) in small intestinal content compared to pork diet [27]. Bai et al. found that bitter melon could improve the metabolic status in high-fat-diet-induced obese mice by means of ^1^ H-NMR, mainly linked to several metabolic patterns with a few effective metabolites, namely lactate, taurine, proline, glucose, ribose, and cholate [28]. Barouei et al. examined the intestinal and systemic responses to incorporating a type 2 resistant starch into a high-fat diet fed to obese mice. They found that predominant among the metabolite differences in the cecum and colon were high quantities of glucose and maltose in mice fed with high-fat and type 2-resistant starch. On the opposite, cecal lactate and butyrate amounts were significantly reduced [29].

To the best of our knowledge, most studies have focused on the changes in microbiota and bile acids metabolism after fiber supplementation, while the systematical metabolomic characterization of cecum contents is rare. In order to fill such a gap, the aim of this study was to investigate the metabolic characteristics of cecum contents in high-fat-diet-induced obese mice supplemented with single or mixed fibers (AX, XG, BG, and inulin) by metabolomics observations based on ^1^ H-NMR. This study could provide hints on the regulatory mechanism of fibers in high-fat-diet-induced obese mice and shed light on the complex interactions between the gut microbial co-metabolism and the host.

## 2. Materials and Methods

### 2.1. Experimental Design

The experimental designs and protocols were all approved by the Animal Ethics Committee of Sichuan Agricultural University (No. DKYB20140302).

**Figure 1:**
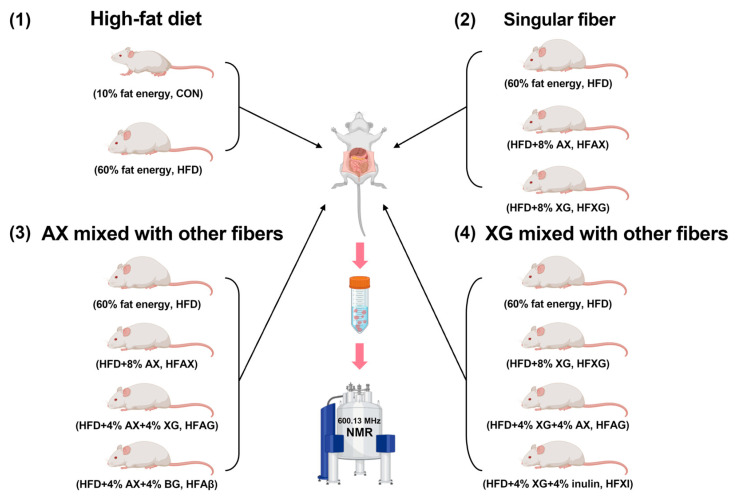
A scheme of experimental design.

Twenty-eight 6-week-old male ICR/KM mice were housed in separate cages through an adaptive feeding period of 1 week, and then, they were randomly divided into seven groups: normal-diet group (10% fat energy, CON), high-fat diet group (60% fat energy, HFD), high-fat-diet group complemented with AX (8% AX, HFAX), high-fat-diet group complemented with XG (8% XG, HFXG), high-fat-diet group complemented with AX and BG (4% AX + 4% BG, HFAβ), high-fat-diet group complemented with AX and XG (4% AX + 4% XG, HFAG), and high-fat-diet group complemented with XG and inulin (4% XG + 4% inulin, HFXI), as shown in Figure 1. After 8 weeks, the mice were fasted for 12 h, then sacrificed by cervical dislocation, and their abdominal cavity was immediately opened. The contents of the cecum were collected and kept in a refrigerator at −80 °C for analysis.

### 2.2. Metabolomic Analysis

Following a previous study [30], we prepared a D_2_O solution for NMR analysis, containing the sodium salt of 3-(trimethylsilyl)-propionic-2,2,3,3-d_4_ acid (TSP, 10 mmol/L), a 1 mol/L phosphate buffer and NaN_3_ (2 mmol/L) in. TSP and NaN_3_ were included in the solution to serve as an NMR spectra chemical-shift reference and against microbial proliferation, respectively. The pH of the solution was then adjusted at 7.00 ± 0.02. To prepare the samples for analysis, we took 100 mg of cecum contents and 1 mL of deionized water to an Eppendorf tube. After vortexing for 5 min, the mixture was centrifuged for 15 min at 18,630× *g* and 4 °C. Then, 700 μL of the supernatant was mixed with 200 μL of NMR analysis solution, and the resulting mixture was centrifuged again at the above conditions. Finally, 650 μL of supernatant was transferred to an NMR tube.

To acquire the ^1^ H-NMR spectra of the samples, we used a 600.13 MHz AVANCE III spectrometer (Bruker, Milan, Italy) at 298 K. To suppress the signals from broad resonances due to large molecules, we employed a CPMG filter composed of 400 echoes with a τ of 400 μs and a 180° pulse of 24 μs, for a total filter of 330 ms, as suggested by Zhu et al. [31]. We also suppressed the HOD residual signal by means of presaturation using the cpmgpr1d sequence. Each spectrum was obtained by summing up 256 transients using 32 K data points over a 7184 Hz spectral window, with an acquisition time of 2.28 s and a recycle delay of 5 s.

The acquired spectra were manually adjusted for phase using Topspin (version 4.2), and subsequent adjustments were made using custom R language scripts. The residual water signal was removed, and baseline correction was performed on the ^1^ H-NMR spectra using the “rolling ball” principle implemented in the baseline R package. To account for differences in water and fiber contents among samples, probabilistic quotient normalization (PQN) was applied to the entire array of spectra. The assignment of compounds was generated by comparing their chemical shift and multiplicity with standard compound spectra in the Chenomx software library (Chenomx Inc., Edmonton, AB, Canada, ver 8.4).

### 2.3. Statistical Analysis

R computational language was used to perform statistical analysis. Before the univariate analyses, the distribution of molecule concentrations was brought to normality in agreement with Box and Cox [32]. We used *t*-test and ANOVA to look for molecules with different concentrations in different groups and then used the Tukey HSD post hoc test (*p* < 0.05).

With the aim of obtaining an overall view of the metabolomes’ trends, robust principal component analysis (rPCA) models were setup on the basis of the molecules accepted by *t*-test and ANOVA analysis. Scoreplot and Pearson correlation plot were calculated for each rPCA model in order to obtain the overall data and to find out the relations between model components and the molecule concentrations.

Enrichment analysis was performed using MetaboAnalyst 5.0. The result of the enrichment analysis highlighted the pathways mainly altered. For this purpose, we only considered molecules with significantly different concentrations in the univariate analysis.

## 3. Results

### 3.1. H-NMR Spectra of Cecum Contents

**Figure 2:**
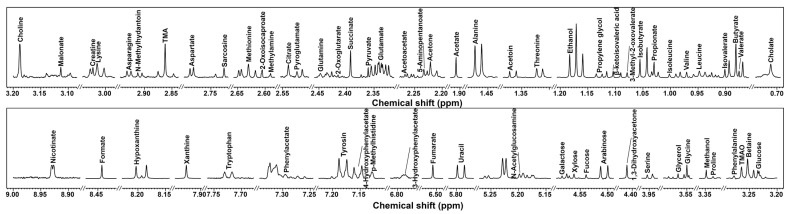
^1^ H-NMR spectrum of cecum contents in mice. Molecule names that appear over the signal were used for their quantifications. For each portion, a spectrum with a convenient signal-to-noise ratio has been selected in order to ease the reader’s visual inspection.

In the present study, by means of ^1^ H-NMR, we were able to identify and quantify 66 metabolites in the cecum contents of mice, providing information about diet, protein digestion, energy production, or gut microbial co-metabolism. A typical ^1^ H-NMR spectrum of the cecum contents is shown in Figure 2.

### 3.2. Effect of High-Fat Die on the Cecum Content Metabolome

Eight of the quantified molecules were significantly different among the CON and HFD groups, namely 3-hydroxyphenylacetate, acetate, acetone, arabinose, methanol, methionine, N-methylhydantoin, and sarcosine, as shown in Table 1.

**Figure 3:**
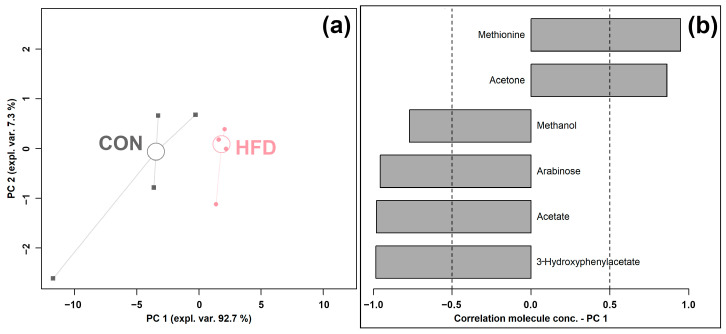
rPCA model was performed on the basis of molecules whose concentrations showed significant differences between CON and HFD groups. Scoreplot (**a**) shows the overall structure of the data, and the loading plot (**b**) shows the significant relationships between the concentration of each molecule and its importance over PC 1.

An rPCA model was generated based on the concentrations of molecules, which were selected by *t*-test, as shown in Figure 3.

As shown in Figure 3a, PC 1 accounted for as much as 92.7% of the entire samples’ variability represented by the model, nicely summarizing the changes between the groups. In detail, the cecum contents of the HFD group were found to be mainly characterized by lower levels of methanol, arabinose, acetate, and 3-hydroxyphenylacetate and a higher level of methionine and acetone compared to the control CON group.

### 3.3. Effect of Single Fiber (AX and XG) on the Cecum Metabolome

**Figure 4:**
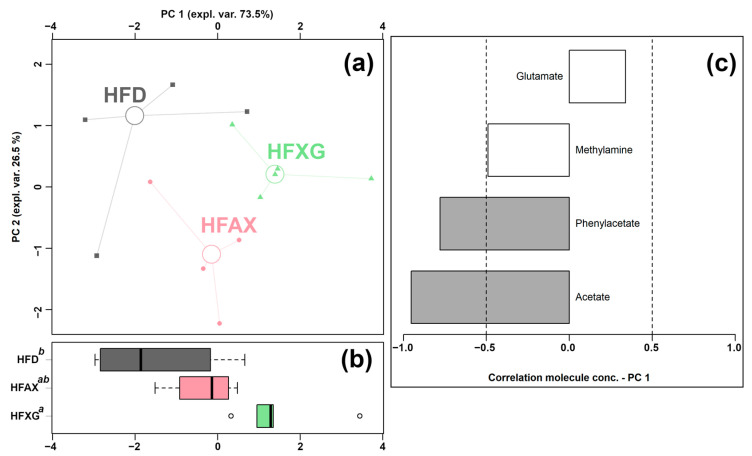
rPCA model was performed on the basis of molecules whose concentrations showed significant differences among HFD, HFXG, and HFAX groups. Scoreplot (**a**) shows the overall structure of the data, boxplot (**b**) highlights the position of the samples along PC 1, and loading plot (**c**) shows the relationships between the concentration of each molecule and its importance over PC 1. Gray bars highlight significant correlations (*p* < 0.05).

Four of the quantified molecules were significantly different among the HFD, HFAX, and HFXG groups, namely acetate, glutamate, methylamine, and phenylacetate, as shown in Table 2. Similar to the above condition, an rPCA model was generated, as shown in Figure 4.

As shown in Figure 4a, PC 1 accounted for as much as 73.5% of the entire samples’ variability represented by the model, nicely summarizing the changes among the groups. In detail, the cecum contents of the HFAX and HFXG groups were found to be mainly characterized by lower levels of methylamine, propionate, and acetate and a higher level of glutamate compared to the control HFD group.

### 3.4. Effect of AX in Combination with Different Fibers (BG and XG) on the Cecum Metabolome

**Figure 5:**
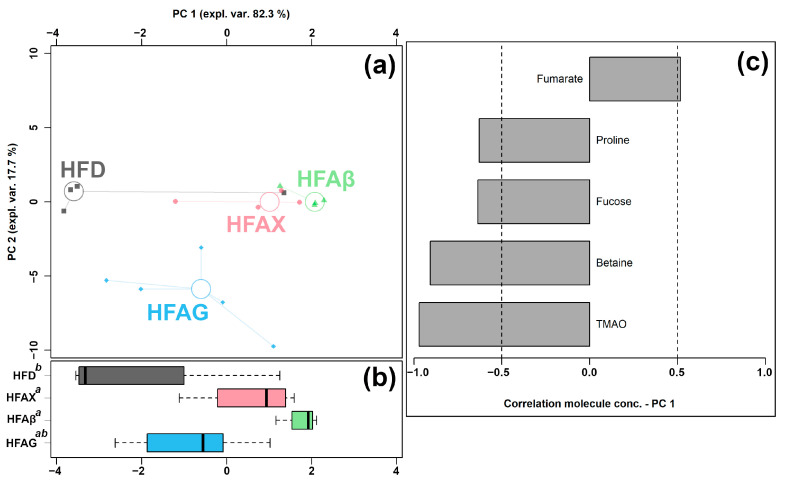
rPCA model was performed on the basis of molecules whose concentrations showed significant differences among HFD, HFAG, HFAX, and HFAβ groups. Scoreplot (**a**) shows the overall structure of the data and boxplot (**b**) highlights the position of the samples along PC 1. The comparisons among the groups are represented by a compact letter display, where group names followed by a common superscript identify no significant differences. Loading plot (**c**) shows the significant relationships between the concentration of each molecule and its importance over PC 1 (*p* < 0.05).

Eight of the quantified molecules were significantly different among the HFD, HFAX, HFAβ, and HFAG groups, namely acetate, betaine, fucose, fumarate, glutamate, N-acetyglucosamine, proline, and TMAO, as shown in Table 3. Similar to the above condition, an rPCA model was generated, as shown in Figure 5.

As shown in Figure 5a, PC 1 accounted for as much as 82.3% of the entire samples’ variability represented by the model, nicely summarizing the differences among the samples from the four groups. In detail, the cecum contents of the HFAX, HFAβ, and HFAG groups were found to be mainly characterized by lower levels of proline, fucose, betaine, and TMAO and by a higher level of fumarate, compared to the control HFD group.

### 3.5. Effect of XG Combined with Different Fibers (AG and Inulin) on the Cecum Metabolome

**Figure 6:**
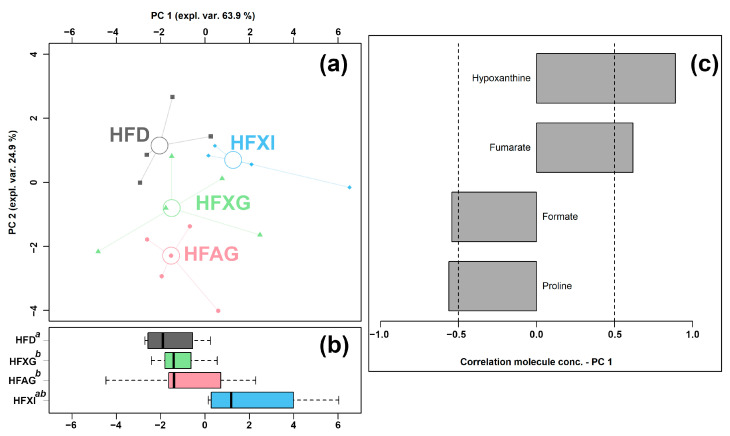
rPCA model was performed on the basis of molecules whose concentrations showed significant differences among HFD, HFAG, HFXG, and HFXI groups. Scoreplot (**a**) shows the overall structure of the data, boxplot (**b**) highlights the position of the samples along PC 1, and loading plot (**c**) shows the significant relationships between the concentration of each molecule and its importance over PC 1 (*p* < 0.05).

Six of the quantified molecules were significantly different among the HFD, HFAG, HFXG, and HFXI groups, namely acetate, formate, fumarate, hypoxanthine, N-acetylglucosamine, and proline, as shown in Table 4. Similar to the above condition, an rPCA model was generated, as shown in Figure 6.

The PC 1 of its scoreplot (Figure 6a) accounted for 63.9% of the entire samples’ variability represented by the model. In detail, the cecum contents of the HFAG, HFXG, and HFXI groups were found to be mainly characterized by lower levels of formate and proline and by higher levels of fumarate and hypoxanthine compared to the control HFD group.

### 3.6. Enrichment Analysis

**Figure 7:**
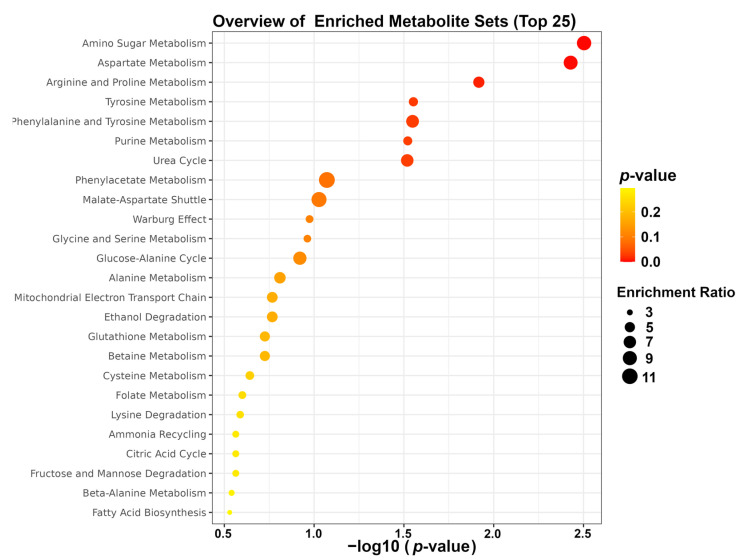
Enrichment analysis.

To gain more in-depth insight into the most extensive changes in the metabolic pathways of high-fat-diet-induced obese mice following fibers supplementation, enrichment analysis was performed by means of the MetaboAnalyst platform. The result of enrichment analysis highlighted that the mainly altered pathways were amino sugar metabolism, aspartate metabolism, and arginine and proline metabolism, as shown in Figure 7.

## 4. Discussion

Recently, dietary interventions have become one of the most accepted ways to prevent obesity and its complications. Compared to drug therapies, dietary interventions are more suitable for all populations because of their low cost and few side effects. Among the various dietary intervention approaches, fiber administration is widely adopted due to its ability to reduce risks of lipid disorders, inflammation, endotoxemia, and dysbiosis of gut bacteria caused by high-fat diets [3]. The obesity-regulating mechanisms of fibers have been studied from several perspectives, such as plasma metabolome [33], urinary metabolome [34], and serum metabolome [35]. However, metabolites of cecum content can effectively capture the complex interactions between the gut microbiome and the host [36], while its metabolomic features were rarely investigated, and a gap remains in the research field [37]. In order to fill this gap, the current study was to comprehensively characterize the metabolome of the cecum and tried to investigate the regulatory effects of single or mixed fibers (AX, XG, BG, and inulin) on high-fat-diet-induced obesity in mice by untargeted metabolomics based on ^1^ H-NMR.

In the present study, higher levels of glutamate, fumarate, and hypoxanthine, as well as lower levels of methylamine, phenylacetate, acetate, fucose, formate, proline, betaine, and TMAO, were found in the high-fat-diet-induced obese mice supplemented with fibers, compared to control subjects. In mammals, glutamate has been reported to be associated with several physiological aspects, such as cell proliferation, biosynthesis of neurotransmitters and other amino acids, immune function, acid-base balance, and gene expression [38]. Moreover, glutamate can be involved in renal gluconeogenesis [38] or the tricarboxylic acid cycle (TCA cycle) under specific metabolic conditions (e.g., nutritional or metabolic stress) [39]. A low level of glutamate has been found in the serum and urine of obese mice [40,41], which suggested that obesity could decrease glutamate concentration in the body. In our study, a higher level of glutamate was found in obese mice supplemented with fibers, which may indicate the beneficial effects of fibers on the cecum metabolome. Fumarate provides information about energy production and is an intermediate in the TCA cycle and the urea cycle [42]. Maulidiani et al. found that obese mice had reduced levels of fumarate in urine, which may be associated with mitochondrial dysfunction [43]. Moreover, significantly downregulated fumarate levels were found in the serum of obese mice, and its concentration could be upregulated when obese mice were supplemented with *Corydalis bungeana* extracts, with anti-obesity effects [44]. Combined with our result, we can infer that fibers may have a positive effect on the regulation of mitochondrial functions, thus regularizing energy metabolism in obese mice. Hypoxanthine is an intermediate metabolite of purine nucleotides, as well as an upstream metabolite of xanthine [45]. In purine metabolism, hypoxanthine is transformed into xanthine by xanthine oxidase (XOD) and finally into uric acid. In our study, a higher level of hypoxanthine was found in obese mice supplemented with fibers, which was in line with previous studies [46]. Such phenomenon might be linked to the inhibition of XOD activity by fibers administration [47].

Methylamine occurs endogenously from amine catabolism. Lavallee et al. found that dysbiosis of the gut microbiota in obese patients could promote the conversion of choline to methylamine [48]. Our previous study revealed that fiber supplementation could improve gut microbiota conditions in obese mice [6,17,18]. In the current study, we found that supplementation with fibers granted significantly lowered levels of methylamine in obese mice, indicating that fibers, in case of obesity, could have beneficial effects on the regulations of gut microbiota, as observed from a metabolomic point of view. Such findings were consistent with Zhang et al., who found that methylamine concentration was significantly reduced in obese mice after fish oil supplementation [49]. Phenylacetate is usually derived from microbial metabolism in mammals. Ji et al. found that *Rosa Roxburghii* Tratt juice (RRTJ) with lipid-lowering effects could have effects on the downregulation of phenylacetate in the feces of obese rats. Moreover, RRTJ treatment reversed the high-fat-diet-induced gut dysbiosis by reducing the presence of *Dorea* and *Coprobacillus* and by increasing the concentration of *Bifidobacterium* and *Roseburia* [50]. Similarly to phenylacetate, increased excretion of fucose induced by a high-fat diet is often associated with dysbiosis of the gut microbiota [51], so the lower levels of phenylacetate and fucose we found in the present study could be linked to gut microbiota regulation. In mammalians, formate is a metabolic mediator among organisms, diet, and gut microbiota. Liu et al. found that plasma formate concentration was positively correlated with body weight and BMI [52]. In humans, obese adults have higher plasma proline levels compared to normal-weight adults [53,54]. Furthermore, Almanza-Aguilera et al. found that a low-energy diet could significantly reduce plasma proline levels in obese individuals [24]. TMAO is a metabolite produced by gut microbiota. Elevated circulating TMAO level is associated with obesity [55]. Hui et al. found that the *Firmicutes/Bacteroidetes* ratio was positively correlated with plasma TMAO concentration [56]. Interestingly, in our previous study, we found that fiber supplementation could reduce the ratio of *Firmicutes/Bacteroidetes* in obese mice, probably leading to the TMAO decline. In addition, enrichment analysis highlighted three main pathways linked to NSP supplementation, namely amino sugar metabolism, aspartate metabolism, and arginine and proline metabolism. Our previous research confirmed that gut microbiota could be altered by different NSP supplementation. Therefore, SCFAs and amino acids, which are the functional output of the gut microbiota, could be modulated underlying the NSPs supplementations. In particular, arginine and proline metabolism could be associated with obesity, resistance to insulin, and lipid levels [57].

## 5. Conclusions

To the best of our knowledge, the present study, for the first time, has been devoted to obtaining a holistic metabolomic representation of obese mice cecum content by providing quantitative information through untargeted ^1^ H-NMR. A total of 66 molecules were identified and quantified. Taking advantage of uni/multivariate analysis, a list of molecules was found to distinguish high-fat-diet-induced obese mice supplemented with single/mixed fibers from control mice, mainly gut microbiota co-metabolites. Enrichment analysis highlighted that the pathway mainly altered was amino acid metabolism. In summary, fiber supplementation could change the metabolomic profiles of cecum contents in obese mice induced by the high-fat diet. Furthermore, mixed NSPs exhibited more beneficial effects than singular form on gut metabolism.

## Figures and Tables

**Table 1:** Molecules with significant differences in cecum contents of CON and HFD groups.

	CON	HFD	*p* Value	Trend
3-Hydroxyphenylacetate	1.87 × 10^−4^ ± 8.15 × 10^−5^	4.69 × 10^−5^ ± 1.28 × 10^−5^	0.0017	↓
Acetate	3.94 × 10^−2^ ± 1.10 × 10^−2^	2.00 × 10^−2^ ± 3.04 × 10^−3^	0.0077	↓
Acetone	2.68 × 10^−5^ ± 2.08 × 10^−5^	6.52 × 10^−5^ ± 9.25 × 10^−6^	0.0266	↑
Arabinose	1.09 × 10^−3^ ± 1.27 × 10^−3^	1.16 × 10^−4^ ± 4.41 × 10^−5^	0.0147	↓
Methanol	7.83 × 10^−4^ ± 2.06 × 10^−4^	1.92 × 10^−4^ ± 2.06 × 10^−4^	0.0067	↓
Methionine	3.81 × 10^−4^ ± 5.55 × 10^−5^	4.87 × 10^−4^ ± 1.89 × 10^−5^	0.0260	↑
N-Methylhydantoin	5.30 × 10^−5^ ± 9.35 × 10^−6^	1.81 × 10^−5^ ± 6.64 × 10^−6^	0.0013	↓
Sarcosine	2.60 × 10^−4^ ± 1.27 × 10^−4^	9.84 × 10^−5^ ± 7.13 × 10^−5^	0.0418	↓

**Table 2:** Molecules with significant differences in cecum contents of HFD, HFAX, and HFAG groups.

	HFD	HFAX	HFXG
Acetate	2.04 × 10^−2^ ± 3.58 × 10^−3 *a*,^*	1.62 × 10^−2^ ± 3.02 × 10^−3 *ab*^	1.25 × 10^−2^ ± 4.23 × 10^−3 *b*^
Glutamate	2.41 × 10^−3^ ± 4.96 × 10^−4 *a*^	1.61 × 10^−3^ ± 3.42 × 10^−4 *b*^	2.37 × 10^−3^ ± 3.10 × 10^−4 *a*^
Methylamine	2.31 × 10^−5^ ± 1.37 × 10^−5 *ab*^	2.89 × 10^−5^ ± 9.76 × 10^−6 *b*^	1.42 × 10^−5^ ± 4.76 × 10^−6 *a*^
Phenylacetate	2.27 × 10^−4^ ± 1.09 × 10^−4 *a*^	1.43 × 10^−4^ ± 4.02 × 10^−5 *ab*^	9.79 × 10^−5^ ± 3.26 × 10^−5 *b*^

* For each molecule, the comparisons among the groups are represented by a compact letter display, where sd values followed by a common superscript identify no significant differences.

**Table 3:** Molecules with significant differences in cecum contents of HFD, HFAX, HFAβ, and HFAG groups.

	HFD	HFAX	HFAβ	HFAG
Acetate	2.04 × 10^−2^ ± 3.58 × 10^−3 *a*,^*	1.62 × 10^−2^ ± 3.02 × 10^−3 *ab*^	9.62 × 10^−3^ ± 1.25 × 10^−3 *b*^	9.13 × 10^−3^ ± 5.64 × 10^−3 *b*^
Betaine	2.59 × 10^−4^ ± 1.49 × 10^−4 *a*^	1.47 × 10^−4^ ± 6.54 × 10^−5 *ab*^	5.84 × 10^−5^ ± 2.10 × 10^−5 *b*^	1.52 × 10^−4^ ± 7.50 × 10^−5 *ab*^
Fucose	1.01 × 10^−4^ ± 4.49 × 10^−5 *ab*^	9.26 × 10^−5^ ± 4.33 × 10^−5 *ab*^	4.43 × 10^−5^ ± 4.90 × 10^−6 *a*^	1.04 × 10^−4^ ± 4.18 × 10^−5 *b*^
Fumarate	8.70 × 10^−5^ ± 3.96 × 10^−5 *b*^	1.52 × 10^−4^ ± 7.42 × 10^−5 *ab*^	4.52 × 10^−4^ ± 3.23 × 10^−4 *a*^	2.21 × 10^−4^ ± 6.58 × 10^−5 *a*^
Glutamate	2.41 × 10^−3^ ± 4.96 × 10^−4 *ab*^	1.61 × 10^−3^ ± 3.42 × 10^−4 *b*^	2.46 × 10^−3^ ± 5.24 × 10^−4 *a*^	2.25 × 10^−3^ ± 1.58 × 10^−4 *ab*^
N-Acetylglucosamine	3.42 × 10^−4^ ± 1.01 × 10^−4 *b*^	3.68 × 10^−4^ ± 6.99 × 10^−5 *b*^	2.44 × 10^−4^ ± 8.41 × 10^−5 *b*^	1.21 × 10^−3^ ± 3.35 × 10^−4 *a*^
Proline	1.05 × 10^−3^ ± 4.36 × 10^−4 *ab*^	7.03 × 10^−4^ ± 2.05 × 10^−4 *ab*^	5.85 × 10^−4^ ± 9.86 × 10^−5 *b*^	1.25 × 10^−3^ ± 2.99 × 10^−4 *a*^
TMAO	1.40 × 10^−4^ ± 7.57 × 10^−5 *b*^	6.37 × 10^−5^ ± 3.73 × 10^−5 *ab*^	3.03 × 10^−5^ ± 1.27 × 10^−5 *a*^	8.82 × 10^−5^ ± 4.95 × 10^−5 *ab*^

* For each molecule, the comparisons among the groups are represented by a compact letter display, where sd values followed by a common superscript identify no significant differences.

**Table 4:** Molecules with significant differences in cecum contents of HFD, HFAG, HFAXG, and HFXI groups.

	HFD	HFAG	HFXG	HFXI
Acetate	2.04 × 10^−2^ ± 3.58 × 10^−3 *a*,^*	9.13 × 10^−3^ ± 5.64 × 10^−3 *b*^	1.25 × 10^−2^ ± 4.23 × 10^−3 *ab*^	1.15 × 10^−2^ ± 4.88 × 10^−3 *ab*^
Formate	5.55 × 10^−4^ ± 4.41 × 10^−4 *ab*^	6.71 × 10^−4^ ± 3.20 × 10^−4 *b*^	2.33 × 10^−3^ ± 2.88 × 10^−3 *b*^	1.86 × 10^−4^ ± 6.06 × 10^−5 *a*^
Fumarate	8.70 × 10^−5^ ± 3.96 × 10^−5 *b*^	2.21 × 10^−4^ ± 6.58 × 10^−5 *ab*^	2.17 × 10^−4^ ± 1.40 × 10^−4 *ab*^	4.21 × 10^−4^ ± 3.76 × 10^−4 *a*^
Hypoxanthine	2.23 × 10^−4^ ± 1.30 × 10^−4 *b*^	3.52 × 10^−4^ ± 1.53 × 10^−4 *ab*^	4.24 × 10^−4^ ± 1.88 × 10^−4 *ab*^	5.89 × 10^−4^ ± 3.62 × 10^−4 *a*^
N-Acetylglucosamine	3.42 × 10^−4^ ± 1.01 × 10^−4 *b*^	1.21 × 10^−3^ ± 3.35 × 10^−4 *a*^	3.71 × 10^−4^ ± 1.78 × 10^−4 *b*^	2.84 × 10^−4^ ± 7.60 × 10^−5 *b*^
Proline	1.05 × 10^−3^ ± 4.36 × 10^−4 *ab*^	1.25 × 10^−3^ ± 2.99 × 10^−4 *a*^	1.07 × 10^−3^ ± 8.74 × 10^−5 *ab*^	6.85 × 10^−4^ ± 1.54 × 10^−4 *b*^

* For each molecule, the comparisons among the groups are represented by a compact letter display, where sd values followed by a common superscript identify no significant differences.

## Data Availability

The data presented in this study are available on request from the corresponding author.
